# Radiofrequency Ablation on Autonomously Functioning Thyroid Nodules: A Critical Appraisal and Review of the Literature

**DOI:** 10.3389/fendo.2020.00317

**Published:** 2020-05-22

**Authors:** Roberto Cesareo, Andrea Palermo, Valerio Pasqualini, Silvia Manfrini, Pierpaolo Trimboli, Fulvio Stacul, Bruno Fabris, Stella Bernardi

**Affiliations:** ^1^Unit of Metabolic Diseases, S. M. Goretti Hospital, Latina, Italy; ^2^Unit of Endocrinology and Diabetes, Campus Bio-Medico University, Rome, Italy; ^3^Department of Radiology, S. F. Neri Hospital, Rome, Italy; ^4^Clinic for Nuclear Medicine and Competence Centre for Thyroid Disease, Imaging Institute of Southern Switzerland, Ente Ospedaliero Cantonale, Bellinzona, Switzerland; ^5^Faculty of Biomedical Sciences, Università di Lugano (USI), Lugano, Switzerland; ^6^Unit of Radiology (Maggiore Hospital), ASUGI, Trieste, Italy; ^7^Unit of Medicina Clinica (Cattinara Hospital), ASUGI, Trieste, Italy; ^8^Department of Medical Sciences, University of Trieste, Trieste, Italy

**Keywords:** radiofrequency ablation, AFTN, benign thyroid adenoma, guideline, thyroid

## Abstract

**Background:** Thyroid nodules are an extremely common occurrence, as their prevalence in the general population is estimated to range between 50 and 70%. Some of these nodules are autonomously functioning such that they can cause hyperthyroidism over time. In this case, surgery and radioiodine represent the standard of care. Nevertheless, patients might have contraindications or be unwilling to undergo these treatments. Minimally-invasive ultrasound-guided techniques, such as laser and radiofrequency ablation (RFA), have been recently introduced into clinical practice as an alternative treatment for symptomatic benign thyroid nodules. Due to their efficacy and tolerability, these techniques have become increasingly available and their usage has been extended also to autonomously functioning thyroid nodules (AFTN).

**Methods:** In this narrative review, we will describe the studies reporting the therapeutic effects of RFA on AFTN, the studies reporting how RFA compares to the other treatment modalities, as well as the current indications for the use of RFA in patients with AFTN. For this purpose, a comprehensive literature search was independently conducted by three investigators on PubMed, EMBASE, and the Cochrane Library from inception up to February 2020 to identify published articles concerning the effects of RFA on AFTN.

**Results and Conclusions:** Current consensus statements and guidelines support the notion that RFA should be regarded as a first-line therapy for non-functioning benign thyroid nodules, while it remains a valid second-line option for AFTN treatment in case of contraindications or patient unwillingness to undergo surgery or radioiodine.

## Introduction

Thyroid nodules are an extremely common occurrence. Some of them are autonomously functioning thyroid nodules (AFTN), which are characterized by the production of a greater amount of thyroid hormones, causing hyperthyroidism over time. The prevalence of AFTN varies according to the geographical area and iodine intake. In the general population, it is estimated that their prevalence ranges from 2.7 to 4.4% (1), but moving from iodine sufficient to iodine deficient areas such as South America and Europe, AFTN can account for as much as 30% of cases of hyperthyroidism (2). Iodine deficiency, which impairs thyroid hormone synthesis and stimulates cellular growth, is the major risk factor for thyroid nodule autonomy development (2). In addition, many AFTN exhibit a somatic point mutation in the *TSHR* gene leading to a constitutive activation of TSHR (3), with subsequent tissue enlargement and thyroid hormone overproduction. Although AFTN can produce greater amounts of thyroid hormones, causing hyperthyroidism over time, many do so slowly, as shown in a study on euthyroid patients with AFTN, who became hyperthyroid at a rate of 4% per year (4). Nevertheless, nodules with a maximum diameter >3 cm (or with a volume >5 ml) are more likely to cause hyperthyroidism over time (5).

Given that antithyroid drugs can reduce thyroid hormones but can not cure AFTN, surgery and radioactive iodine represent the standard of care for this condition (6), particularly when AFTN become symptomatic (7). Nevertheless, patients might have contraindications or be unwilling to undergo these procedures. Recently, minimally-invasive ultrasound-guided techniques, such as laser and radiofrequency ablation (RFA), have been introduced into clinical practice as an alternative treatment for symptomatic benign thyroid nodules (8), including AFTN (9–11).

Focusing on RFA, this is an outpatient procedure, which is generally performed under local anesthesia. It requires the ultrasound-guided insertion of an electrode needle generating an alternating electric field into the nodule. Then, the electrode tip is sequentially moved from the deepest to the superficial parts of the nodule, inducing rapid heating of the target zones. Treatment is accompanied by the formation of coagulative necrosis, and, over time, by fibrotic changes with progressive nodule shrinkage (12, 13). The use of RFA to treat symptomatic thyroid nodules is supported by robust evidence of efficacy and tolerability (14), with a risk of major complications lower than 1% (15).

Here, we will review the therapeutic effects of RFA on AFTN, how this technique compares to the other existing procedures (surgery and radioiodine) in terms of efficacy, complications, and cost, as well as the current indications to RFA in patients with AFTN. For this purpose, a comprehensive literature search was independently conducted by three investigators on PubMed, EMBASE, and the Cochrane Library from inception up to February 2020 to identify published articles concerning the effects of RFA on AFTN. The term radiofrequency was matched with the following terms: thyroid, hyperthyroidism, thyrotoxicosis, autonomous, or toxic or hyperfunctioning adenoma, or autonomously functioning thyroid nodules. We searched for articles published in English and those involving human participants. Additional exclusion criteria for full texts included pediatric populations and case reports. Three investigators independently searched papers, screened titles, and abstracts of the retrieved articles, reviewed the full-texts and selected articles for their inclusion.

### Therapeutic Effects of Radiofrequency Ablation on AFTN

Since the first report on the efficacy of RFA in reducing benign thyroid nodules (16), several works have evaluated whether RFA could normalize thyroid function as well (Table 1) (17–24). Overall, in these works, despite the average volume reduction was always >50%, the rate of thyroid function normalization at last follow-up was extremely heterogeneous, ranging from 24 to 86%. This variability has been ascribed to technical reasons given that in the first studies (17, 19, 20) the procedure was carried out with the multiexpandable needle, while in the most recent ones it was carried out with the moving shot technique and the 18-G needle. In addition, in some of these studies, patients underwent more than one RFA session (18, 19, 22). Moreover, these studies had a retrospective design, a short follow-up period, a small sample size, and they included AFTN together with non-functioning thyroid nodules.

**Table 1:** Summary of studies evaluating RFA efficacy on AFTN.

**Author** **(references)**	**Study design**	**Mean patient** **age** **(years)**	**No. AFTN (AFTN/total nodules)**	**Electrode type**	**No. of sessions**	**Previous** **treatments** **(no. of pts)**	**Therapy** **with** **ATD**	**Nodule type**	**Mean initial volume** **(mL)**	**Follow-up** **duration** **(months)**	**Mean volume reduction rate** **at last follow-up**	**Percentage of patients with thyroid function normalization** **at last follow-up**
Deandrea et al. (17)	Prospective	67	23/31	14-G	1	–	All	Solid/mixed	27.7	6	50.7%	24%
Baek et al. (18)	Retrospective	47	9/9	17-G and 18-G	1–4	–	2/9	Solid/mixed/cystic	14.9	6	70.7%	56%
Spiezia et al. (19)	Prospective	72.5	28/94	14-G	1–3	Surgery (9) Radioiodine (12)	All	Solid	24.5	24	79.4%	79%
Faggiano et al. (20)	Prospective	58	10/20	14-G	1	Surgery (2) Radioiodine (2)	All	Solid	13.3	12	86%	40%
Sung et al. (22)	Retrospective	43	44/44	18-G	1–6	–	5/44	Solid/mixed	18.5	6	74.5%	82%
Bernardi et al. (21)	Prospective	69	30/30	18-G	1	–	All	Solid/mixed	17.1	12	75%	50%
Cesareo et al. (23)	Prospective	51	29/29	18-G	1	–	None	Solid	5 (A) 18 (B)	24	84% (A) 68% (B)	86% (A) 45% (B)
Dobnig and Amrein (24)	Prospective	52	32/277	18-G	1	–	17/32	Solid/mixed	Not specified for AFTN	3–12	N/A	84.3%

Nodule volume (V) and Volume reduction (Vreduction) were calculated by the following formulas: V = πabc/6 (where V is the volume, a is the maximum diameter, and b and c are the other two perpendicular diameters) and Vreduction = (initial–finalV/initialV) ^*^ 100.

*Thyroid function normalization was defined as TSH normalization (within reference ranges) without anti-thyroid drugs*.

*(A) is for small AFTN group; (B) is for medium size AFTN group; N/A is for not applicable*.

To overcome these limitations, our groups designed two separate prospective studies. The first study (21) aimed at evaluating the 12-month efficacy of a single session of RFA performed with the moving shot technique. In this work, RFA was performed on 30 AFTN, with an average volume of 17 mL. After 12 months, RFA reduced thyroid nodule volume by 75%, leading to thyroid function normalization in 50% of patients. Interestingly, in this study, patients went into remission when their nodules were reduced on average by 81% after 12 months from the procedure. Patients who improved, in comparison, had their nodules reduced by 68%. The second study aimed at comparing the response to RFA of small AFTN (<12 mL) to that of medium size AFTN (>12 mL) (23). Interestingly, in this work, the rate of thyroid function normalization significantly increased in small nodules (whose average volume was 5 mL) as compared to medium size nodules (whose average volume was 18 mL). Specifically, the rate of thyroid function normalization was 86% in small nodules vs. 45% in medium size nodules. This was consistent with the rate of nodules converting from hot to cold at the scintiscan, which was 86% in small nodules as compared to 16% in medium size nodules. In addition, small size nodules exhibited a greater volume reduction, as they were reduced by 82 and 84% after 12 and 24 months, as compared to medium size nodules, which were reduced by 67 and 68% after 12 and 24 months. These data indicate that small AFTN have a more favorable outcome. In addition, also in this study there was a higher probability of symptom resolution and thyroid function normalization when the volume ablation was >80%, which is in line with the concept that the greater the baseline volume the higher the likelihood to undertreat hyperfunctioning areas, leading to hyperthyroidism/symptom relapse.

Most of these studies (17–24) have been included in a recent metanalysis (25) evaluating the efficacy of RFA in AFTN in terms of TSH normalization, scintiscan changes, and volume reduction rate. Overall, this metanalysis included 8 articles and 205 AFTN treated with RFA. Five studies used a single session of RFA. Follow-up ranged from 6 to 24 months. This metanalysis showed that the pooled rate of TSH normalization was 57%, the pooled rate of scintiscan changes was 60%, and the pooled volume reduction at 1 year was 79%. Interestingly, also in this metanalysis baseline nodule volume was associated with the rate of TSH normalization.

### Comparison Between Radiofrequency Ablation and the Other Techniques for the Treatment of AFTN

Only a few studies comparing RFA to surgery or to radioiodine have been published so far. In addition, the studies comparing RFA to surgery either included AFTN together with non-functioning nodules (26–28), or did not specify the functional status of the nodules (29). In the first study comparing RFA to surgery (hemithyroidectomy) (26), there were no differences between the techniques in terms of improvement of nodule-related symptoms and cosmetic concerns, while RFA was significantly less effective than surgery in terms of thyroid function normalization in the subgroup of patients with AFTN. Surgery was not as well tolerated as RFA, and the cost of one session of RFA was €1,661.50 as compared to conventional hemithyroidectomy whose cost was €4,556.30 and short-stay hemithyroidectomy whose cost was €4,139.40, which is consistent with the costs reported by other Authors (30). In 2017, the same group conducted a telephone survey in 115 patients treated with RFA and 68 patients treated with hemithyroidectomy to enquire about their satisfaction after 12 months from either procedure. While in the subgroup of patients with non-functioning nodules these techniques did not differ, in the subgroup of patients with AFTN, RFA was not as fully satisfactory as surgery in terms of resolution of nodule-related symptoms (27).

As mentioned above, other groups have compared RFA to surgery. In particular, Che et al. (28) reported that RFA had lower postoperative medication use, lower hypothyroidism, and lower complication rate than surgery. Nevertheless, this is a study where both lobectomy and total thyroidectomy were included in the surgery group, which might not be the appropriate comparison group for RFA, given that the ideal candidate of RFA is a patient with a single benign thyroid nodule (31), who should be generally treated with lobectomy (and not thyroidectomy). In the study of Yue et al., where RFA was compared to lobectomy, the Authors found that RFA was associated with a significantly better health-related quality of life (HRQoL) scores in terms of mental general health, vitality, and mental health after 6 months from the treatment. Nevertheless, it has to be taken into account that surgery requires at least 1 year for a full recovery. On the other hand, RFA was found more expensive than surgery, possibly because all patients were hospitalized (29).

Traditionally, radioiodine is the main alternative to surgery for AFTN treatment, due to its efficacy and low cost (32). So far, only one study has compared RFA to radioiodine in AFTN (33). In this retrospective study, nodule volume was reduced by 76% after 12 months from RFA, and by 68% after 12 months from radioiodine. Interestingly, euthyroidism restoration was achieved in 90.9% of patients with RFA and 72% of patients with radioiodine. These percentages of thyroid function normalization are higher than those reported by other authors (21). This could be ascribed—at least in part—to the baseline volume of the nodules (23), which was 14 mL.

Besides RFA, the ultrasound-guided technique that has been recently used for AFTN is laser ablation (34). Although there are no studies comparing RFA to laser ablation in terms of AFTN management, the direct comparison of the two techniques has been the focus of recent studies reaching different conclusions (35–39). In this context, the only randomized clinical trial comparing RFA to laser ablation showed that technique efficacy was achieved in 86.7% of patients treated with RFA as compared to 66.7% of patients treated with laser ablation, and that RFA was associated with a significantly greater nodule volume reduction after 6 months from the procedure (38).

### Consensus Statements and Guidelines on the Use of Radiofrequency Ablation for the Treatment of AFTN

The first recommendations on RFA for treating benign thyroid nodules were released by the Korean Society of Radiology in 2012. In these recommendations, the indications to RFA included symptoms, cosmetic concerns, and thyrotoxicosis due to a benign thyroid nodule (9). A few years later, in 2015, a panel of several Italian scientific societies advised the use of RFA in case of either large non-functioning benign thyroid nodules or AFTN, when surgery and radioiodine were contraindicated or declined (10). Interestingly, in this consensus statement, the Authors put forward the potential usefulness of combining RFA with radioiodine for the treatment of large toxic goiters, based on the promising results obtained combining laser therapy with radioiodine (40), as this would increase nodule volume shrinkage and allow for a reduction of the radioiodine dose.

Then, in 2016, the AACE updated its guidelines on thyroid nodule and it included RFA as a treatment option for solid or complex nodules that progressively enlarged, were symptomatic, or caused cosmetic concern (8). Likewise, also the European Federation of Societies for Ultrasound in Medicine and Biology (EFSUMB) produced a set of recommendations on the use of ultrasound-guided procedures, highlighting the concept that RFA should be considered as an alternative to surgery or radioiodine for symptomatic benign thyroid nodules (41).

It is only recently that consensus statements and guidelines have differentiated the indications to RFA between patients with non-functioning nodules and patients with AFTN, suggesting that RFA should be a first-line option for non-functioning nodules and a second-line option for AFTN. In particular, in the last revision of the Korean Society of Radiology recommendations (11), benign thyroid nodules causing local symptoms or cosmetic concerns were considered as a strong recommendation to the procedure, while AFTN only a weak recommendation. In addition, this guideline differentiated the number of cytology reports of benignity requested prior to the procedure, because in case of non-functioning nodules the panel recommended two benign cytology reports, while in case of AFTN only one report, due to the fact that AFTN are generally considered benign lesions (6). However, given that AFTN show a wide morphologic spectrum of follicular neoplasms (42) and they may fall into the cytological category of indeterminate/follicular lesions, it remains to be clarified if, in such case, they can be equally treated with RFA. Recently, the Italian Minimally-Invasive Treatment of the Thyroid (MITT) group has produced a consensus statement, which is in line with the 2017 Korean guidelines. In particular, in case of AFTN, before the procedure it is required only one cytology report of benignity. In addition, RFA can be proposed as a first-line treatment for non-functioning thyroid nodules, while in case of AFTN it represents only a therapeutic option when conventional treatments are refused or contraindicated. Moreover, as compared to the Korean guidelines, the MITT group consensus statement has introduced the concept that, when advising patients, it is important to consider that the best responses have been observed in small size AFTN, and that the highest probability of symptom resolution and thyroid function normalization is when the nodule is reduced by 80% (43).

## Discussion/Conclusions

In conclusion, the literature shows that RFA normalizes thyroid function in 45–50% of medium size AFTN and in more than 80% of small size AFTN. This is associated with a significant nodule volume reduction after 24 months from the treatment, ranging from 68 to 84%. Only a few low quality studies have compared RFA to other techniques. However, if RFA does not seem to perform as well as surgery, at least in terms of symptom control in patients with AFTN, the comparison between RFA and radioiodine showed promising results.

Consistent with the available literature, current consensus statements, and guidelines support the notion that RFA effectively reduces thyroid nodule volume and improves local symptoms, while it does not always normalize thyroid function. In particular, while RFA is regarded as a first-line therapy for non-functioning benign thyroid nodules, it remains a valid second-line option for AFTN treatment in case of contraindications or unwillingness to undergo surgery or radioiodine. In addition, it has to be taken into account that AFTN are more likely to respond in terms of volume reduction and function normalization, when their baseline volume is <12 mL and when volume is reduced by at least 80% after 12 months from the treatment.

Prospective studies with larger sample sizes to evaluate RFA effects and randomized controlled trials comparing RFA to surgery and radioiodine are needed to confirm and improve our current understanding of RFA on AFTN.

## Author Contributions

RC, SB, AP contributed to conception and design of the review and carried out an independent comprehensive literature search. RC, AP, VP, SM, PT, FS, BF, and SB wrote the first draft of the manuscript, wrote sections of the manuscript, contributed to manuscript revision, and approved it for publication.

## Conflict of Interest

FS is a consultant of H.S. Hospital Service S.p.A. (LT), Italy. The remaining authors declare that the research was conducted in the absence of any commercial or financial relationships that could be construed as a potential conflict of interest.

## References

[1] Belfiore A Sava L Runello F Tomaselli L Vigneri R. Solitary autonomously functioning thyroid nodules and iodine deficiency. J Clin Endocrinol Metab. (1983) 56:283–7. 10.1210/jcem-56-2-283 6822638

[2] Baltisberger BL Minder CE Burgi H. Decrease of incidence of toxic nodular goitre in a region of Switzerland after full correction of mild iodine deficiency. Eur J Endocrinol. (1995) 132:546–9. 10.1530/eje.0.1320546 7749493

[3] Krohn K Fuhrer D Bayer Y Eszlinger M Brauer V Neumann S. Molecular pathogenesis of euthyroid and toxic multinodular goiter. Endocr Rev. (2005) 26:504–24. 10.1210/er.2004-0005 15615818

[4] Sandrock D Olbricht T Emrich D Benker G Reinwein D. Long-term follow-up in patients with autonomous thyroid adenoma. Acta Endocrinol (Copenh). (1993) 128:51–5. 10.1530/acta.0.1280051 8447194

[5] Hegedus L Bonnema SJ Bennedbaek FN. Management of simple nodular goiter: current status and future perspectives. Endocr Rev. (2003) 24:102–32. 10.1210/er.2002-0016 12588812

[6] Haugen BR Alexander EK Bible KC Doherty GM Mandel SJ Nikiforov YE. 2015 American Thyroid Association Management guidelines for adult patients with thyroid nodules and differentiated thyroid cancer: the American Thyroid Association guidelines task force on thyroid nodules and differentiated thyroid cancer. Thyroid. (2016) 26:1–133. 10.1089/thy.2015.0020 26462967 PMC4739132

[7] Thomas CG Jr Croom RD 3rd. Current management of the patient with autonomously functioning nodular goiter. Surg Clin North Am. (1987) 67:315–28. 10.1016/S0039-6109(16)44186-1 3551148

[8] Gharib H Papini E Garber JR Duick DS Harrell RM Hegedus L. American Association of Clinical Endocrinologists, American College of Endocrinology, and Associazione Medici Endocrinologi Medical Guidelines for Clinical Practice for the diagnosis and management of thyroid nodules−2016 update. Endocr Pract. (2016) 22:622–39. 10.4158/EP161208.GL 27167915

[9] Na DG Lee JH Jung SL Kim JH Sung JY Shin JH. Radiofrequency ablation of benign thyroid nodules and recurrent thyroid cancers: consensus statement and recommendations. Korean J Radiol. (2012) 13:117–25. 10.3348/kjr.2012.13.2.117 22438678 PMC3303894

[10] Garberoglio R Aliberti C Appetecchia M Attard M Boccuzzi G Boraso F. Radiofrequency ablation for thyroid nodules: which indications? The first Italian opinion statement. J Ultrasound. (2015) 18:423–30. 10.1007/s40477-015-0169-y 26550079 PMC4630279

[11] Kim JH Baek JH Lim HK Ahn HS Baek SM Choi YJ. 2017 Thyroid radiofrequency ablation guideline: Korean society of thyroid radiology. Korean J Radiol. (2018) 19:632–55. 10.3348/kjr.2018.19.4.632 29962870 PMC6005940

[12] Bernardi S Stacul F Zecchin M Dobrinja C Zanconati F Fabris B. Radiofrequency ablation for benign thyroid nodules. J Endocrinol Invest. (2016) 39:1003–13. 10.1007/s40618-016-0469-x 27098804

[13] Cesareo R Palermo A Pasqualini V Cianni R Gaspa G Manfrini S. Radiofrequency ablation for the management of thyroid nodules: a critical appraisal of the literature. Clin Endocrinol (Oxf). (2017) 87:639–48. 10.1111/cen.13422 28718950

[14] Cesareo R Pasqualini V Simeoni C Sacchi M Saralli E Campagna G. Prospective study of effectiveness of ultrasound-guided radiofrequency ablation versus control group in patients affected by benign thyroid nodules. J Clin Endocrinol Metab. (2015) 100:460–6. 10.1210/jc.2014-2186 25387256

[15] Kim C Lee JH Choi YJ Kim WB Sung TY Baek JH. Complications encountered in ultrasonography-guided radiofrequency ablation of benign thyroid nodules and recurrent thyroid cancers. Eur Radiol. (2017) 27:3128–37. 10.1007/s00330-016-4690-y 27975148

[16] Kim YS Rhim H Tae K Park DW Kim ST. Radiofrequency ablation of benign cold thyroid nodules: initial clinical experience. Thyroid. (2006) 16:361–7. 10.1089/thy.2006.16.361 16646682

[17] Deandrea M Limone P Basso E Mormile A Ragazzoni F Gamarra E US-guided percutaneous radiofrequency thermal ablation for the treatment of solid benign hyperfunctioning or compressive thyroid nodules. Ultrasound Med Biol. (2008) 34:784–91. 10.1016/j.ultrasmedbio.2007.10.018 18207307

[18] Baek JH Moon WJ Kim YS Lee JH Lee D. Radiofrequency ablation for the treatment of autonomously functioning thyroid nodules. World J Surg. (2009) 33:1971–7. 10.1007/s00268-009-0130-3 19575141

[19] Spiezia S Garberoglio R Milone F Ramundo V Caiazzo C Assanti AP. Thyroid nodules and related symptoms are stably controlled two years after radiofrequency thermal ablation. Thyroid. (2009) 19:219–25. 10.1089/thy.2008.0202 19265492

[20] Faggiano A Ramundo V Assanti AP Fonderico F Macchia PE Misso C. Thyroid nodules treated with percutaneous radiofrequency thermal ablation: a comparative study. J Clin Endocrinol Metab. (2012) 97:4439–45. 10.1210/jc.2012-2251 23019349

[21] Bernardi S Stacul F Michelli A Giudici F Zuolo G de Manzini N. 12-month efficacy of a single radiofrequency ablation on autonomously functioning thyroid nodules. Endocrine. (2017) 57:402–8. 10.1007/s12020-016-1174-4 27848197

[22] Sung JY Baek JH Jung SL Kim JH Kim KS Lee D. Radiofrequency ablation for autonomously functioning thyroid nodules: a multicenter study. Thyroid. (2015) 25:112–7. 10.1089/thy.2014.0100 25320840

[23] Cesareo R Naciu AM Iozzino M Pasqualini V Simeoni C Casini A. Nodule size as predictive factor of efficacy of radiofrequency ablation in treating autonomously functioning thyroid nodules. Int J Hyperthermia. (2018) 34:617–23. 10.1080/02656736.2018.1430868 29357717

[24] Dobnig H Amrein K. Monopolar Radiofrequency ablation of thyroid nodules: a prospective austrian single-center study. Thyroid. (2018) 28:472–80. 10.1089/thy.2017.0547 29490593 PMC5905420

[25] Cesareo R Palermo A Benvenuto D Cella E Pasqualini V Bernardi S Efficacy of radiofrequency ablation in autonomous functioning thyroid nodules. A systematic review and meta-analysis. Rev Endocr Metab Disord. (2019) 20:37–44. 10.1007/s11154-019-09487-y 30887407

[26] Bernardi S Dobrinja C Fabris B Bazzocchi G Sabato N Ulcigrai V. Radiofrequency ablation compared to surgery for the treatment of benign thyroid nodules. Int J Endocrinol. (2014) 2014:934595. 10.1155/2014/934595 25045352 PMC4090443

[27] Bernardi S Dobrinja C Carere A Giudici F Calabro V Zanconati F. Patient satisfaction after thyroid RFA versus surgery for benign thyroid nodules: a telephone survey. Int J Hyperthermia. (2018) 35:150–8. 10.1080/02656736.2018.1487590 30107758

[28] Che Y Jin S Shi C Wang L Zhang X Li Y. Treatment of benign thyroid nodules: comparison of surgery with radiofrequency ablation. AJNR Am J Neuroradiol. (2015) 36:1321–5. 10.3174/ajnr.A4276 25814656 PMC7965284

[29] Yue WW Wang SR Li XL Xu HX Lu F Sun LP. Quality of life and cost-effectiveness of radiofrequency ablation versus open surgery for benign thyroid nodules: a retrospective cohort study. Sci Rep. (2016) 6:37838. 10.1038/srep37838 27883069 PMC5121639

[30] Filetti S Ladenson PW Biffoni M D'Ambrosio MG Giacomelli L Lopatriello S. The true cost of thyroid surgery determined by a micro-costing approach. Endocrine. (2017) 55:519–29. 10.1007/s12020-016-0980-z 27172916

[31] Lupo MA. Radiofrequency ablation for benign thyroid nodules–a look towards the future of interventional thyroidology. Endocr Pract. (2015) 21:972–4. 10.4158/EP15797.CO 26247236

[32] Patel NN Abraham P Buscombe J Vanderpump MP. The cost effectiveness of treatment modalities for thyrotoxicosis in a U.K. center. Thyroid. (2006) 16:593–8. 10.1089/thy.2006.16.593 16839261

[33] Cervelli R Mazzeo S Boni G Boccuzzi A Bianchi F Brozzi F. Comparison between radioiodine therapy and single-session radiofrequency ablation of autonomously functioning thyroid nodules: a retrospective study. Clin Endocrinol (Oxf). (2019) 90:608–16. 10.1111/cen.13938 30657603

[34] Pacella CM Mauri G. Is there a role for minimally invasive thermal ablations in the treatment of autonomously functioning thyroid nodules? Int J Hyperthermia. (2018) 34:636–8. 10.1080/02656736.2018.1462537 29793352

[35] Ha EJ Baek JH Kim KW Pyo J Lee JH Baek SH. Comparative efficacy of radiofrequency and laser ablation for the treatment of benign thyroid nodules: systematic review including traditional pooling and bayesian network meta-analysis. J Clin Endocrinol Metab. (2015) 100:1903–11. 10.1210/jc.2014-4077 25695887

[36] Pacella CM Mauri G Cesareo R Paqualini V Cianni R De Feo P. A comparison of laser with radiofrequency ablation for the treatment of benign thyroid nodules: a propensity score matching analysis. Int J Hyperthermia. (2017) 33:911–9. 10.1080/02656736.2017.1332395 28605944

[37] Mauri G Cova L Monaco CG Sconfienza LM Corbetta S Benedini S. Benign thyroid nodules treatment using percutaneous laser ablation (PLA) and radiofrequency ablation (RFA). Int J Hyperthermia. (2017) 33:295–9. 10.1080/02656736.2016.1244707 27701923

[38] Cesareo R Pacella CM Pasqualini V Campagna G Iozzino M Gallo A. Laser ablation versus radiofrequency ablation for benign non-functioning thyroid nodules: six-month results of a randomized, parallel, open-label, trial (LARA Trial). Thyroid. (2020). 10.1089/thy.2019.0660. [Epub ahead of print]. 32056501

[39] Trimboli P Castellana M Sconfienza LM Virili C Pescatori LC Cesareo R. Efficacy of thermal ablation in benign non-functioning solid thyroid nodule: a systematic review and meta-analysis. Endocrine. (2020) 67:35–43. 10.1007/s12020-019-02019-3 31327158

[40] Chianelli M Bizzarri G Todino V Misischi I Bianchini A Graziano F. Laser ablation and 131-iodine: a 24-month pilot study of combined treatment for large toxic nodular goiter. J Clin Endocrinol Metab. (2014) 99:E1283–6. 10.1210/jc.2013-2967 24684455

[41] Dietrich CF Bamber J Berzigotti A Bota S Cantisani V Castera L EFSUMB Guidelines and recommendations on the clinical use of liver ultrasound elastography, update 2017 (long version). Ultraschall Med. (2017) 38:e16–e47. 10.1055/s-0043-103952 28407655

[42] Harach HR Sanchez SS Williams ED. Pathology of the autonomously functioning (hot) thyroid nodule. Ann Diagn Pathol. (2002) 6:10–9. 10.1053/adpa.2002.30605 11842375

[43] Papini E Pacella CM Solbiati LA Achille G Barbaro D Bernardi S. Minimally-invasive treatments for benign thyroid nodules: a Delphi-based consensus statement from the Italian minimally-invasive treatments of the thyroid (MITT) group. Int J Hyperthermia. (2019) 36:376–82. 10.1080/02656736.2019.1575482 30909759

